# Integrative medicine during antepartum hospitalization: A single‐center retrospective pre‐post study

**DOI:** 10.1002/pmf2.70372

**Published:** 2026-07-25

**Authors:** Hadley Ross, Casandra Chouravong, Ana Vera, Ariana Braddom, Adah Beck, Jan E. Patterson, Angela Boyd

**Affiliations:** ^1^ Department of Obstetrics and Gynecology UT Health San Antonio San Antonio Texas USA; ^2^ Integrative Medicine Program University Health San Antonio Texas USA; ^3^ University of Texas Health Science Center San Antonio Texas USA; ^4^ Department of Internal Medicine UT Health San Antonio San Antonio Texas USA

**Keywords:** aromatherapy, antepartum hospitalization, high‐risk pregnancy, integrative medicine, mind‐body therapies, patient‐reported discomfort

## Abstract

**Objective:**

Integrative medicine (IM) therapies are increasingly used alongside conventional medical care to address physical discomfort, stress, sleep disruption, and difficulty relaxing. However, IM remains understudied among hospitalized antepartum patients. We evaluated patterns of IM use and short‐term changes in discomfort scores among hospitalized antepartum patients receiving IM consultation.

**Study design:**

We conducted a single‐center retrospective pre‐post study of antepartum patients admitted to a tertiary care center who received at least one IM consultation between January 2024 and July 2025. IM therapies included aromatherapy, meditation and breathwork, sound therapy, and relaxation techniques. Because IM consultations were provided at patient/provider request, the study population reflects patients selected for or interested in IM services rather than all hospitalized antepartum patients. Patient‐reported discomfort was assessed immediately before and after each IM encounter by the IM nurse using a five‐point ordinal scale from 0 to 4, with 0 indicating no discomfort and 4 indicating severe discomfort. For patients with multiple IM visits, only one paired pre/post discomfort score was included in the paired analysis, prioritizing the first IM visit with complete pre‐ and post‐intervention discomfort scores. Encounters without both pre‐ and post‐intervention discomfort scores were excluded from paired analysis. Paired scores were compared using the Wilcoxon signed‐rank test.

**Results:**

A total of 207 antepartum patients received IM consultations during the study period, and 174 patients had paired pre‐ and post‐intervention discomfort scores available for analysis. Mean gestational age at admission was 30 weeks, and median length of stay was 7 days. The most common admission indication was hypertensive disorders of pregnancy (31%). Aromatherapy (94%) and mind–body therapies, including meditation and breathwork (51%), were the most frequently used IM modalities. Median discomfort scores decreased from 2 before IM to 1 after IM (*p* < 0.0001). The median paired change was −1 (Interquartile range [IQR] −1 to 0). Discomfort scores decreased in 130 patients (75%), remained unchanged in 44 patients (25%), and none reported increased discomfort.

**Conclusion:**

Among hospitalized antepartum patients who received IM consultation, aromatherapy and mind–body therapies were the most frequently used modalities. IM encounters were associated with short‐term reductions in patient‐reported discomfort scores; however, given the pre‐post design and the absence of a control group, these findings should be interpreted as hypothesis‐generating rather than causal.

## 1 INTRODUCTION

Integrative medicine (IM) combines conventional medical care with evidence‐based complementary therapies to support physical, emotional, and psychological well‐being. Common IM modalities include aromatherapy, mindfulness, breathing techniques, relaxation therapy, sound therapy, and other mind–body interventions. These approaches are increasingly incorporated into healthcare to support symptom management, stress reduction, comfort, relaxation, sleep, and quality of life [1, 2].

Use of IM is common among women of reproductive age, many of whom seek non‐pharmacologic strategies during pregnancy [3]. Prior obstetric research has largely focused on IM use during labor, delivery, and the postpartum period. In these settings, mind–body therapies and aromatherapy have been associated with reductions in anxiety, stress, pain perception, physical discomfort, sleep disturbance, fatigue, and other pregnancy‐related symptoms [4, 5, 6, 7, 8]. Despite growing interest, the role of IM earlier in pregnancy, particularly among hospitalized antepartum patients, remains poorly characterized.

Antepartum hospitalization often involves prolonged admission for high‐risk pregnancy conditions and may be associated with physical discomfort, emotional distress, social isolation, restricted mobility, disrupted sleep, and prolonged separation from usual routines. Prior studies suggest IM therapies may reduce physical discomfort, pain, stress, anxiety, sleep disturbance, and fatigue [4, 5, 6, 7, 8, 9]. Discomfort during pregnancy is multidimensional and may include pain, fatigue, musculoskeletal symptoms, sleep disturbance, restlessness, and other symptoms that affect daily function [10, 11, 12]. However, few studies have evaluated IM use or short‐term symptom response among hospitalized antepartum patients.

The objective of this study was to characterize patterns of IM use and to assess short‐term within‐encounter changes in patient‐reported discomfort scores among hospitalized antepartum patients receiving IM services.

## 2 METHODS

We conducted a single‐center retrospective pre‐post study of hospitalized antepartum patients at a tertiary care academic medical center between January 2024 and July 2025. The institutional IM service provided non‐pharmacologic therapies to inpatients upon provider or patient request. This study was reviewed and approved by the Institutional Review Board. Verbal consent for IM services was obtained from the patient prior to the consultation.

All obstetric patients admitted to the antepartum service during the study period were eligible for inclusion if they received at least one IM consultation during hospitalization. IM consultations were requested at the discretion of providers, nurses, or patients; therefore, patients included in this analysis represent a patient/provider‐selected subgroup of the broader antepartum inpatient population. There were no restrictions based on gestational age, admission diagnosis, or length of hospitalization.

Each IM consultation was considered a clinical encounter during which one or more IM modalities could be delivered. For analyses describing patient characteristics and IM use patterns, patients were counted once, regardless of the number of IM visits received during the study period. For the paired analysis of discomfort scores, only one paired pre‐ and post‐intervention discomfort score was included per patient. When patients had multiple IM encounters, the first IM visit with complete pre‐ and post‐intervention discomfort scores was prioritized. Encounters without both pre‐ and post‐intervention discomfort scores were excluded from the paired analysis. Because this was an observational study of routinely delivered inpatient services, no additional interventions or follow‐up procedures were performed beyond standard clinical care.

IM therapies were delivered by a single nurse who is a board‐certified holistic nurse with certifications in aromatherapy, mindfulness‐based stress reduction, sound therapy, and somatic trauma therapy. Aromatherapy was administered through nasal inhalation, topical application with appropriate dilution, transdermal delivery using a premade patch, or passive diffusion. Additional modalities included mind–body therapies such as meditation, guided breathwork, stress‐reduction techniques, sleep and relaxation interventions, sound therapy, and color/light therapy. Multiple modalities were frequently provided during a single visit based on patient preference and practitioner assessment.

Data abstracted from the electronic medical record included maternal age, gravidity, parity, gestational age at admission and delivery, admission indication, mode of delivery, length of stay, and number of IM visits. Patient‐reported discomfort was assessed immediately before and immediately after the IM consultation by the nurse providing the interventions using a five‐point ordinal scale, with 0 indicating no discomfort and 4 indicating severe discomfort. Discomfort was used instead of stress or anxiety because of the heterogeneous symptoms prompting IM consultation. Common reasons for referral to IM included pain, anxiety, stress, and insomnia. Thus, this pragmatic scale is routinely used by the IM consult service to assess symptom‐burden response to holistic interventions. Validated pregnancy‐specific discomfort, pain, anxiety, or symptom‐burden instruments were not collected as part of the usual IM care during the study period.

Descriptive statistics were used to summarize patient characteristics and IM use. The number of IM visits per patient was summarized using median and interquartile range. Because discomfort scores were paired, ordinal, and limited to five possible values, pre‐ and post‐intervention discomfort scores were compared using the Wilcoxon signed‐rank test. Results were also summarized using the median paired change and the number and percentage of patients whose scores improved, remained unchanged, or worsened. All tests were two‐sided, with *p* < 0.05 considered statistically significant. Statistical analyses were performed using GraphPad Prism version 11.0.0 for Mac, GraphPad Software, Boston, Massachusetts, USA.

## 3 RESULTS

A total of 207 hospitalized antepartum patients received at least one IM consultation during the study period. Baseline demographic and clinical characteristics are summarized in (Table 1). The mean maternal age was 31.1 ± 6.6 years. Median gravidity and parity were 2 and 1, respectively. Mean gestational age at admission was 30.1 weeks, and mean gestational age at delivery was 32.9 weeks. The most common admission indication was hypertensive disorders of pregnancy (31%), followed by preterm prelabor rupture of membranes (19%). The median length of hospital stay was 7 days. Most patients received one to two IM visits during hospitalization. Most patients delivered during the admission, with cesarean delivery more common than vaginal delivery (49% vs. 29%). Table 1.

**TABLE 1:** Characteristics of integrative medicine patients.

Variable	Value (*n* = 207)
Age, years	31.1 ± 6.6
Gravidity	2 (1–4)
Parity	1 (0–2)
Gestational age at admission	30.1 ± 6.0
Gestational age at delivery	32.9 ± 4.7
Mode of delivery	
Cesarean delivery	48.7% (*n* = 101)
Vaginal delivery	28.9% (*n* = 60)
Undelivered	22.2% (*n* = 46)
Primary admission indication	
High blood pressure	31.4% (*n* = 65)
Rupture of membranes	19.3% (*n* = 40)
Non‐reassuring fetal status	6.8% (*n* = 14)
Placenta issues	9.7% (*n* = 20)
Preterm labor	6.8% (*n* = 14)
Maternal complications	11.1% (*n* = 23)
Other	14.9% (*n* = 31)
Length of admission, days	7 (4–16)
Number of integrative medicine visits	1 (1–2)

**TABLE 2:** Integrative medicine therapies.

Variable	Value (*n* = 207)
Integrative therapies	
Aromatherapy	93.7% (*n* = 194)
Passive diffuser	28.9% (*n* = 60)
Nasal	60.9% (*n* = 126)
Topical	55.1% (*n* = 114)
Transdermal	57.9% (*n* = 120)
Mind–body therapy	50.7% (*n* = 105)
Sleep and relaxation	40.6% (*n* = 84)
Sound therapy	6.3% (*n* = 13)
Color and light therapy	6.3% (*n* = 13)

IM sessions frequently included more than one modality. Aromatherapy was the most commonly used modality (94%), administered via nasal inhalation, topical application, transdermal delivery, or passive diffusion (Table 2). A lavender‐peppermint blend was commonly used. Mind–body therapies, including meditation and guided breathwork, were used in 51% of encounters, while sleep and relaxation interventions were used in 41%. No IM‐related adverse events were documented.

Among the 174 IM patients with paired pre‐ and post‐intervention discomfort scores available, the median discomfort score decreased from 2 before IM (Interquartile range [IQR] 2–3) to 1 after IM (IQR 1–2) (*p* < 0.0001, Table 3). The median paired change in discomfort score was −1 point (IQR −1 to 0). Discomfort scores decreased in 130 patients (75%), remained unchanged in 44 patients (25%), and none reported increased discomfort.

**TABLE 3:** Change in patient‐reported discomfort scores after IM consultation.

Measure	Result
Patients with pre‐ and post‐discomfort scores, *n*	174
Pre‐IM discomfort score, median (IQR)	2 (2–3)
Post‐IM discomfort score, median (IQR)	1 (1–2)
Paired change in discomfort score, median (IQR)	−1 (−1 to 0)
Wilcoxon signed‐rank test *p* value	<0.0001

Direction of change	*n* (%)
Decreased discomfort score	130 (75%)
No change in discomfort score	44 (25%)
Increased discomfort score	0 (0%)

Abbreviations: IM, Integrative medicine; IQR, Interquartile range.

## 4 DISCUSSION

In this single‐center retrospective pre‐post study of hospitalized antepartum patients who received IM consultation, IM encounters were associated with short‐term reductions in patient‐reported discomfort scores. Discomfort scores decreased in 75% of patients, with a median one‐point improvement, 25% reported no change, and none reported worsening of discomfort. Aromatherapy and mind–body techniques were the most frequently delivered modalities and were often used in combination. Because all patients received IM and no comparison group was available, these findings demonstrate a temporal association between IM encounters and lower post‐session discomfort scores rather than a causal treatment effect.

Although the median paired decrease was modest, the direction and consistency of improvement support the potential value of IM as a supportive adjunct during antepartum hospitalization. This finding is clinically relevant because pregnancy‐related discomfort is multidimensional and may include pain, fatigue, sleep disturbance, musculoskeletal symptoms, restlessness, and difficulty relaxing [10, 11, 12].

Our findings add to prior studies of IM in obstetrics, which have primarily focused on labor, delivery, or the postpartum period rather than the antepartum inpatient setting. Non‐pharmacologic interventions such as mindfulness, relaxation techniques, and aromatherapy have been associated with reductions in physical discomfort, sleep disturbance, fatigue, and other pregnancy‐related symptoms in pregnant and postpartum populations [4, 5, 6, 7, 8]. However, limited data describe how these therapies are used among patients hospitalized earlier in pregnancy. This study helps characterize real‐world IM use patterns and short‐term symptom changes among hospitalized antepartum patients.

Antepartum hospitalization is often prolonged and occurs in the setting of high‐risk pregnancy conditions, which may contribute to physical discomfort, emotional distress, disrupted routines, restricted mobility, and social isolation. Because many pregnant patients seek non‐pharmacologic approaches to symptom relief, particularly when concerns about medication exposure are present, IM may represent an appealing adjunct to standard inpatient obstetric care [3, 4]. Aromatherapy and mind–body techniques are generally considered low‐risk when delivered by trained practitioners and can be incorporated within routine inpatient obstetric care workflows without disrupting standard obstetric management [5]. These therapies may support relaxation and coping during prolonged hospitalization.

Strengths of this study include the relatively large sample size and the evaluation of IM therapies as they were delivered in real‐world inpatient clinical practice. By examining a multimodal, individualized IM service in routine care, our findings reflect the feasibility and patterns of IM use within a tertiary obstetric hospital environment.

This study has several important limitations. The retrospective, single‐center pre‐post design without a control group limits generalizability and precludes causal inference. The discomfort scale was a routinely documented clinical score rather than a validated patient‐reported outcome measure, and it captured a broad symptom domain. Because the scale had only five possible values, ties and unchanged scores were expected. In addition, part of the observed improvement may reflect the supportive nature of the IM visit itself, including time, attention, reassurance, and therapeutic presence, rather than the independent effect of a specific modality. Finally, validated pregnancy‐specific discomfort or symptom‐burden instruments were not collected, including tools designed to assess pregnancy‐related discomforts and the functional impact of pregnancy symptoms [10, 11].

Future studies should evaluate IM therapies among hospitalized antepartum patients using validated patient‐reported outcome measures, including pregnancy‐related discomfort, pain, symptom burden, sleep quality, anxiety, and patient satisfaction. Prospective studies with comparison groups or randomized designs are needed to clarify whether changes in discomfort are attributable to specific IM modalities, the supportive therapeutic encounter, or other aspects of inpatient care. Future work should also assess patient satisfaction, length of stay, neonatal outcomes, and cost.

## 5 CONCLUSION

Among a patient/provider‐selected group of hospitalized antepartum patients, IM encounters were associated with short‐term reductions in patient‐reported discomfort scores. In a paired analysis using one pre/post score per patient, 75% of patients reported decreased discomfort, 25% reported no change, and none reported worsening after IM. As obstetric care increasingly emphasizes respectful, individualized, and supportive inpatient experiences, IM may serve as a low‐risk adjunct during prolonged high‐risk antepartum hospitalizations. Further prospective studies using validated patient‐reported outcome measures and appropriate comparison groups are warranted to evaluate the broader effects of IM in antepartum inpatient care.

## CONFLICT OF INTEREST STATEMENT

The authors declare no conflicts of interest.

## FUNDING INFORMATION

The authors received no specific funding for this work.

## ETHICS STATEMENT

This study was reviewed and approved by the University of Texas Health San Antonio Review Board and the University Health Institutional Review Board. Given retrospective design and use of existing clinical data, informed consent was waived.
